# The Association between Neighborhood Disorder and Health: Exploring the Moderating Role of Genotype and Marriage

**DOI:** 10.3390/ijerph18030898

**Published:** 2021-01-21

**Authors:** Man-Kit Lei, Ronald L. Simons

**Affiliations:** Department of Sociology, The University of Georgia, Athens, GA 30602, USA; rsimons@uga.edu

**Keywords:** neighborhood disorder, inflammatory burden, physical health, genotype, marital status, African Americans

## Abstract

The present study extends prior research on the link between neighborhood disorder and health by testing an integrated model that combines various social and biological factors. Hypotheses were tested using a sample of 325 African American women from the Family and Community Health Study (FACHS). As expected, inflammatory burden was the biophysiological mechanism that mediated much of the association between neighborhood physical disorder and perceived physical health. This finding provided additional support for the view that global self-ratings of health are powerful predictors of morbidity because, in large measure, they are indicators of chronic, systemic inflammation. Further, both genetic variation and marital status served to moderate the association between neighborhood disorder and health. Finally, being married largely eliminated the probability that neighborhood disorder would combine with genetic vulnerability to increase inflammatory burden and perceived illness. Overall, the findings demonstrate the value of constructing integrated models that specify various biophysiological mechanisms that link social conditions to physical health.

## 1. Introduction

Over the past two decades, there has been increasing interest in how social conditions influence health and disease [1,2,3,4,5]. For example, recent research indicates that lower socioeconomic status (SES) and exposure to childhood adversity are associated with a higher prevalence of chronic illnesses, including diabetes, hypertension, cardiovascular disease, and immune system dysregulation [6,7,8,9,10]. In addition to individual SES, numerous studies have provided evidence suggesting that the neighborhoods where people live are fundamental contexts for understanding physical health [11,12,13,14,15]. In particular, the visible cues of neighborhood disorder (e.g., abandoned buildings, graffiti, and persistently broken windows) represent ambient threats that may lead to various health problems [16]. Building upon this research, the present study is concerned with increasing our understanding of how neighborhood disorder combines with various social and biological processes to foster physical illness. We extend prior research on this issue in several respects.

Most studies utilize self-rated measures of both neighborhood context and general health. Thus, the associations reported in past research are undoubtedly inflated due to shared methods variance [17]. We avoid this problem by using observer ratings of neighborhood disorder and biomarkers of health. In addition to self-rated health, there has been little consideration of the biophysiological mechanisms whereby neighborhood factors influence health. It is generally assumed that adverse conditions such as neighborhood disorder foster physiological stress that, over time, leads to illness [18,19,20]. While this is likely the case, there is a need for research that tests for specific biophysiological mediators [21,22]. Past research has reported that stressful circumstances are associated with elevated circulating markers of inflammation, whereas other studies have shown that elevated levels of inflammation predict virtually all chronic age-related diseases, such as cardiovascular disorders, type II diabetes, osteoporosis, rheumatoid arthritis, Alzheimer’s disease, and certain cancers [23]. Building on these findings, the present study tests the hypothesis that it is chronic inflammation that links adverse neighborhood conditions to risk for illness.

Further, social scientists frequently use global self-ratings to assess health. Although such measures might be viewed as problematic in many respects [24], global self-rated health has repeatedly been shown to predict mortality (after controlling for diagnosed health conditions, depression, and health-related behaviors) and recent evidence indicates that biomarkers of inflammation are associated with self-assessments of health [25]. This pattern of findings suggests that inflammation may be the mediating mechanism linking life stress, such as living in a disordered neighborhood, to self-ratings of health. The present study tests this idea.

Given the modest association that has been shown to exist between stress and physical wellbeing, researchers have turned their attention to individual differences that might account for why some people become ill in the face of adversity whereas others do not. The present study investigates not only the extent to which marital status moderates the impact of neighborhood disorder on inflammation and perceived physical health, but also the extent to which genetic vulnerability amplifies this link. Indeed, little consideration has been given to how genes might moderate the effect of adverse social conditions either on inflammation or self-rated health. These various issues are examined in the present study.

Finally, most neighborhood studies focus on children and adolescents [26,27] and, to a lesser degree, adult men. Females tend to be ignored in neighborhood research even though the impact of neighborhood characteristics on health is more pronounced for women than men [12,28,29,30,31]. Moreover, African Americans are more likely to reside in extremely disordered neighborhoods [32] and display higher rates of morbidity and mortality from chronic disease than other racial groups [33]. Given this, we test our hypotheses using data from a sample of several hundred adult African American women.

### 1.1. Neighborhood Disorder and Health

Prolonged involvement with stressful environments is seen as fostering a physiological stress response that increases the chances of chronic illness by damaging tissue and impairing the immune system [18,19]. In recent years, social scientists have drawn upon the stress process model to develop arguments regarding the role of neighborhood disorder in explaining health inequalities [15,34]. Neighborhood physical disorder refers to visible cues (e.g., graffiti, scattered trash, vandalism, run-down and abandoned buildings) indicating a breakdown of social order and control in an area [12,13,16,28]. Past research indicates that residents find such conditions to be threatening and demoralizing. They are seen as visible cues of ambient threat. Even if residents have not been personally victimized, they perceive a real and persistent danger; people in the neighborhood cannot be trusted, social control no longer exists, and life is essentially chaotic [35]. Consonant with the stress process model [18,19,34], the distress of living under these circumstances is seen as promoting physiological stress and illness [12,16]. Support for this idea has been provided by studies reporting an association between neighborhood disorder and various health outcomes [28].

Although individuals living in disordered neighborhoods appear to be at high risk for chronic illness, several theoretical and methodological issues remain to be addressed. Regarding the latter, neighborhood studies of health tend to assess both neighborhood conditions and health status utilizing respondents’ self-reports. As a consequence, reported associations between neighborhood disorder and disease may be inflated due to “shared methods variance” [17]. To avoid this problem, the present study uses “systematic social observation” [36] to measure objective characteristics present within neighborhoods and both blood-derived biomarkers and self-reports to assess illness. Further, studies of the impact of social disorder on health rarely control for other dimensions of community/neighborhood life, such as neighborhood disadvantage or social isolation. Such controls are necessary in order to establish that the effect of disorder on health is not due to its association with some other characteristic of the area.

Theoretically, while the stress process model assumes that neighborhood stress is linked to illness through the promotion of dysregulated physiological processes, rarely is there any attempt to specify or assess these mediating biophysiological processes. Instead, prior studies have tended to focus on mediating psychological indicators of stress [13,16,37]. In contrast to psychological indicators of distress, physiological manifestations of stress cannot be readily assessed using traditional survey methodology. Although there is evidence that social isolation increases the risk of mortality through elevated inflammatory biomarkers [21,38], few neighborhood studies include assessments of physiological stress-response mechanisms. Indeed, prolonged involvement with adverse environments provides highly salient cues of threat that trigger physiological stress and a cascade of biological responses that, over time, cause wear and tear on physiological systems [14,39]. In the present study, we investigate the hypothesis that elevated inflammatory burden is an important physiological mechanism linking neighborhood stress and physical health.

### 1.2. Biomarkers of Inflammatory Burden

In the past decade, medicine has come to view elevated, systemic inflammation as an important signal of immune dysfunction that portends changes in major physiological systems and development of the age-related diseases that kill most individuals in Western societies [40]. However, the body does not simply respond in this fashion when it has been attacked; it also initiates the inflammatory response when it is threatened or anticipates some type of environmental assault. In support of this idea, there is growing evidence that prolonged exposure to adverse social environments is associated with chronically elevated biomarkers of inflammation. In particular, elevations in C-reactive protein (CRP) and soluble interleukin-6 receptor (sIL-6R) have been associated with chronic illness. Both of these biomarkers are assayed using blood plasma. CRP is a biomarker of vascular and systemic inflammation that is an acute phase serum protein and is produced by the liver. There is evidence that women have higher levels of CRP than men [29,30] and that CRP is a prototypic marker of inflammation and is an important risk factor for diabetes, hypertension, atherosclerosis, and coronary heart disease [41].

The soluble receptor of IL-6 (sIL-6R) is a marker of chronic cytokine activity [42]. Previous studies have indicated that it plays a role in the regulation of IL-6 and is associated with diabetes, chronic illness, certain cancers, and neurological disorders [43,44]. While several studies have focused on the association between each of these inflammatory markers and chronic stress, others have reported that the inflammatory burden, a combination of inflammatory markers, has substantially more variation, predictive ability, and sensitivity to detect the effect of interest [45,46].

Importantly for our purposes, several studies have reported a link between neighborhood characteristics and these biomarkers of inflammation. For example, using a sample of 1410 adults living in Dallas, Browning and colleagues [28] found that men living in neighborhoods with higher burglary rates exhibited elevated CRP levels. Similarly, Broyles et al. [40] reported that children living in disadvantaged or unsafe neighborhoods were more likely to have elevated CRP levels than those living in advantaged or safe neighborhoods, and Gallo et al. [47] found that neighborhood disadvantage is positively related to CRP in Mexican American women. Finally, using samples from unauthorized Brazilian migrants, Holmes and Marcelli [48] also found that neighborhood disorder is positively associated with greater levels of CRP. Consonant with these findings for CRP, research also indicates that living in disordered neighborhoods [49] is associated with elevated sIL-6R levels after controlling for individual characteristics.

In addition to these studies showing a relation between biomarkers of inflammation and neighborhood conditions, there is evidence that these biomarkers are linked to self-ratings of health. As noted above, most neighborhood studies assess health using global self-ratings. A variety of studies have reported that life stress predicts global self-ratings of health [50], suggesting that these self-rating measures may be more a reflection of individuals’ emotional states than of their actual physical health. Contrary to this view, however, self-ratings are stronger predictors of mortality than diagnosed illness [51,52]. This robust link between perceived health and mortality has been puzzling, but clues regarding the possible underlying biophysiological mechanism were recently provided by a study showing an association between self-rated health and biomarkers of inflammation, viz., IL-6 and CRP [25]. Prior research has reported that biomarkers of inflammation are associated with general sickness symptoms, including decreased appetite and fatigue. Together, these studies suggest that inflammation may give rise to feelings of sickness that color a person’s self-rated health. Thus, in the present study, we expect to find that biomarkers of inflammation predict self-rated health and mediate the association between neighborhood disorder and self-rated health.

### 1.3. Genetic Variation and Marital Status as Moderators

According to the classical neighborhood assumption, people from the same neighborhood are more similar to each other than to those from different neighborhoods [53]. However, while individuals living in disadvantaged neighborhoods are more likely to become ill than those residing in more advantaged areas, many, if not most, residents of adverse neighborhoods remain healthy [9,54,55]. This raises the question: Why do some people living in disordered neighborhoods develop serious illnesses whereas others do not? Lei and Simons label this view of multilevel structural resilience [54]. For instance, Lei, Beach, and Simons [15] found that African Americans living in disadvantaged neighborhoods with low perceived collective efficacy in their neighborhood showed significantly accelerated cardiometabolic aging. Thus, resilience mechanisms occur at various levels, ranging from individual to neighborhood [54]. Understanding which individual characteristics influence the relationship between neighborhood contexts and physical health is fundamental to the advancement of neighborhood research.

During the past decade, investigators have turned their attention to the interaction of genes and the environment (G × E) [56,57,58,59,60], and mounting evidence suggests that genetic variation often interacts with environmental stress to influence physical health and inflammatory burden [61,62]. No research, however, has examined the effect of gene by neighborhood interactions on inflammatory burden and health. Given evidence that neighborhood effects on human behavior are often moderated by individual characteristics and experiences [54,63,64] and that genetic differences are a potential source of inflammatory response [9,65], it seems likely that genetic variation may condition the impact of neighborhood adversity on inflammatory burden and physical health.

To address this issue, the present study examines the effect of a single-nucleotide polymorphism (SNP) *rs2228145* (also known as *rs8192284*) in the IL-6 receptor gene. This gene, which is located at the proteolytic cleavage site, results in a substitution of aspartic acid for alanine [66]. Studies have indicated that the minor C allele of *rs2228145* is associated with elevated serum levels of sIL-6R and CRP [67,68,69,70]. This suggests that it may operate as a diathesis that increases vulnerability to stressors such as neighborhood disorder. Accordingly, we hypothesize that people carrying the minor C allele of *rs2228145* will experience an amplification of the probability that neighborhood disorder will lead to an elevated inflammatory burden.

In addition to G × E influences, an individual’s vulnerability to stressors such as neighborhood disorder may be conditioned by access to support. Social scientists have long been interested in the extent to which social support affects health [3,71] and have proposed the stress-buffering hypothesis, which argues that social support buffers the relationship between stress and health [6,72,73]. It is assumed that social support produces this effect because it involves the provision of important emotional and instrumental resources that enhance one’s ability to cope with stressful circumstances. Consonant with this idea, recent neighborhood research has demonstrated that social support buffers the effects of neighborhood disorder or disadvantage on health and wellbeing [37,53].

Of the relationships considered in the stress-buffering literature, marriage has been determined to be one of the most important sources of social support. There is evidence, for example, that married people tend to be in better physical health than unmarried individuals because of their greater access to emotional support, material resources, and social connectedness [74,75,76]. In contrast, unmarried individuals report more psychological distress, have more chronic illnesses [77], and demonstrate higher rates of mortality and morbidity [78] than married persons.

Building on this line of research, several studies have found that marriage is a source of social and emotional support which buffers the relationship between neighborhood disorder and health. This research indicates that the relationship between neighborhood disorder and health problems is stronger for never-married adults than for married adults. However, studies have not investigated the biophysiological mechanisms whereby marriage achieves this effect. The present study examines the extent to which marriages serves to buffer the effect of neighborhood disorder on health by moderating the association between neighborhood disorder and inflammation.

Further, if the neighborhood–gene interaction is correct, it is unclear whether both marital status and the IL-6 receptor gene simultaneously moderate the effect of neighborhood disorder on inflammatory burden. Building on the stress-buffering hypothesis, the current study hypothesizes that the association between neighborhood disorder and inflammatory burden will be strongest among unmarried women carrying the risk allele of *rs2228145*.

### 1.4. Summary of Research Hypotheses

**Figure 1:**
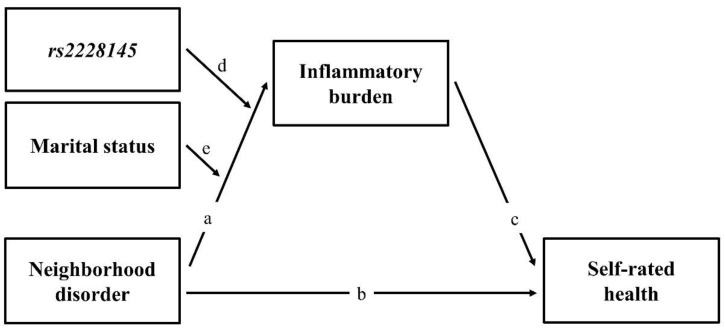
Theoretical model showing hypothesized pathways.

Summarizing, there are two sets of hypotheses for the current study. Figure 1 shows the theoretical model tested in the present study. The first set of hypotheses addresses the relationships among neighborhood disorder, inflammatory burden, and self-rated physical health.

**Hypotheses** **1.** *Neighborhood disorder will be associated with both inflammatory burden and self-rated health (Pathways a and b)*.

**Hypotheses** **2.** *Inflammatory burden will mediate the association between neighborhood disorder and self-rated health (Pathways a and c)*.

The second set of hypotheses addresses the extent to which *rs2228145* and marital status moderate the effect of neighborhood disorder on inflammatory burden and health.

**Hypotheses** **3.** *The association between neighborhood disorder and inflammatory burden will be stronger for individuals who carry the minor C allele of the IL-6 receptor gene than for carriers of the major A allele (Pathway d)*.

**Hypotheses** **4.** *Building upon hypotheses 2 and 3, the interaction effect of neighborhood disorder and the IL-6 receptor gene will have an indirect effect on self-rated health through its effect on inflammatory burden (Pathways d, a, and c)*.

**Hypotheses** **5.** *The association between neighborhood disorder and inflammatory burden will be stronger for unmarried individuals than for those who are married (Pathway e)*.

**Hypotheses** **6.** *Building upon hypotheses 3 and 5, the association between neighborhood disorder and inflammatory burden will be stronger for unmarried carriers of the minor allele of the IL-6 receptor gene than for married persons who carry either the major or minor allele or unmarried persons who carry the major allele (Pathways d and e)*.

**Hypotheses** **7.** *Based on hypotheses 2 and 6, the interaction effect of neighborhood disorder, the IL-6 receptor gene, and marital status will have an indirect effect on self-rated health through its effect on inflammatory burden (Pathways d, e, a, and c)*.

## 2. Materials and Methods

### 2.1. Sample

Data for this study are drawn from the Family and Community Health Study (FACHS), a multi-site investigation of neighborhood and family effects on health and development. FACHS was designed to identify neighborhood and family processes that contribute to the development of African American children [57,60]. The sample strategy was intentionally designed to generate families representing a range of socioeconomic statuses and neighborhood settings. Each family included a child who was in 5th grade at the time of recruitment. At the first wave, the FACHS sample consisted of 889 African American children (411 boys and 478 girls) with their primary caregivers (PCs; 60 men and 829 women). At study inception, around half of the sample resided in Georgia and the other half in Iowa. The children averaged ten years of age (5th grade) at the beginning of the study in 1997–1998. At Wave 1, the sample had an average family per capita income of USD 6956. Thirty six percent of the families were below the poverty line, and fifty one percent of the respondents identified as single parents. Of the 889 PCs interviewed at Wave 1, 693 were interviewed again at Wave 5 (77.26% of the original sample). As part of Wave 5 (2007–2008) data collection, PCs were asked to provide a saliva sample for genotype analysis. Of the 693 participants, 549 (80%) agreed to DNA collection, and a saliva sample was obtained from 472 females. Successful genotyping for inflammation markers was achieved for 460 females (a call rate of 97.5%). Further, roughly 80% of these women agreed to provide blood (*N* = 375).

The current study involves both individual and neighborhood characteristics. The measures of neighborhood characteristics were created using the 2000 Census Summary Tape File 3 (STF3A), which was geocoded with participants’ residential addresses. Additional details regarding neighborhood data can be found in Simons [64] and Lei [57]. The current study is based upon the 325 female respondents who were nested within 88 Census tracts, who agreed to provide a saliva sample and biomarkers, and for whom data on all of the study measures, including systemic social observation neighborhoods data, were available at Wave 5. The resulting sample had a mean age of around 49 years, with a range of 35–65 years. Comparisons of those participants excluded from the present analyses but retained in the sample did not display any significant differences with regard to neighborhood and individual characteristics. To further assess attrition bias, we used Heckman’s two-step procedure to estimate sample selection bias [79]. The results showed that the inverse Mills ratio was not significant and including this ratio parameter in our models did not change the findings. There were no significant differences between those remaining in the panel and those dropping out with regard to a variety of measures such as age, education, income, and mental health. For the 88 Census tracts based on the 2000 Census, 58% of the neighborhoods were urban areas, and 32% had a population more than half of which was African American. The average poverty rate across the 88 Census tracts was 28 percent (*SD* = 0.14).

### 2.2. Procedures

At Wave 5, computer-assisted interviews were administered in the respondent’s home and took on average around two hours to complete. The instruments were presented on laptop computers. Questions appeared in sequence on the screen, which both the researcher and participant could see. The researcher read each question aloud and the participant entered an anonymous response using a separate keypad. When visiting the participant families, two different interviewers also rated the face-block level neighborhoods on physical appearance. The face-block is the unit of observation used in the present study and it was defined by the block segment on the side of the street containing the respondent’s residence [36]. The measures of neighborhood characteristics were created using the 2000 Census data, which were geocoded with participants’ residential addresses at Wave 5. The findings indicated that our respondents had lived in their neighborhoods for an average of over five years.

In addition, participants were also asked to provide a blood sample at Wave 5. Samples were frozen and shipped via courier to a laboratory at the University of Iowa. After blood was drawn into serum separator tubes by certified phlebotomists, it was frozen and shipped via courier to a laboratory at the University of Iowa. Serum levels of CRP and sIL-6R were determined using a Duo Set Kit (DY1707; RandD Systems, Minneapolis, MN, USA) according to the manufacturer’s specifications.

### 2.3. Measures

#### 2.3.1. Dependent Variables

*Inflammatory burden* was measured with two biomarkers of inflammation. C-reactive protein (CRP), a biomarker of vascular and systemic inflammation from a blood sample, was measured at Wave 5. A normal concentration of CRP in healthy human serum is usually lower than 10 mg/L, and CRP levels above 30 mg/L may reflect the presence of an acute infection [48]. Thus, individuals with such high levels of CRP have been excluded (*n* = 5, 1.5%). Because CRP displayed a skewed distribution (skewedness = 2.968, kurtosis = 10.209), it was transformed using log transformation to meet the assumption of linearity for ordinary least squares regression (skewedness = −1.175, kurtosis = 3.207 after the transformation). Soluble IL-6 receptor (sIL-6R), which is the cognate receptor for IL-6, was assayed at Wave 5. Similar to CRP, sIL-6R displayed a skewed distribution (skewedness = 1.881, kurtosis = 9.885). We applied a log transformation to normalize the index (skewedness = −0.005, kurtosis = 2.260 after the transformation). The two biomarkers were significantly correlated (*r* = 0.110, *p* = 0.026). Using the factor analysis, two biomarkers were loaded by the same factor at 0.741, accounting for 54.897% of the reliable variance. Finally, an index of inflammatory burden was calculated by summing the standardized log-transformed biomarker scores for the two measures using the canonical weights method [80].

*Self-rated poor health* status was assessed with an item [81] that asked, “In general, would you say your physical health is…” The response format for the item ranged from 0 (excellent) to 4 (poor). The mean score for this variable was 1.951 (*SD* = 0.986), with roughly 32% of the sample reporting that they were in either poor or fair health.

#### 2.3.2. Independent Variable

*Neighborhood disorder* was assessed using observer ratings of the participants’ neighborhoods as Census data do not provide detailed information about physical signs of incivilities in neighborhoods [82]. Two interviewers visited each family. One conducted the interview with the primary caregivers while the other conducted the interview with the child. Both interviewers were trained in the use of questions from the systemic social observation survey [36]. Prior to entering the respondent’s residence to conduct the interview, they each made an independent, confidential rating of the face-block using three items that focused on physical signs of graffiti, vandalism, and abandoned buildings [13,83]. The rating format for each item ranged from 0 (No, none present) to 3 (Yes, a considerable amount). The intra-class correlation for the two observers was 0.62, indicating good agreement [84]. Scores were summed across items and interviewers to form a measure of neighborhood disorder. Higher scores indicated higher degrees of neighborhood disorder. The measure was standardized (a mean of 0 and a standard deviation of 1).

#### 2.3.3. Moderators

*Marital status* was coded as a binary variable (1 = married; 0 = unmarried). Among the 325 women used in the analysis, 30.5% of subjects reported that they were married at Wave 5.

*Genotype*. All participants were genotyped using TaqmanR MGB assays (Applied Biosystems, Foster City, CA, USA) and the Fluidigm Biomark Genetic Analysis System (Fluidigm, South San Francisco, CA, USA). The SNP *rs2228145* (Asp358Ala) is located on exon 9 of the IL-6r gene on chromosome 1q21. There are three main models for coding gene sequences [85]. Using the dominant model, individuals receive a score of 1 if they are either heterozygous or homozygous for the minor allele and a score of 0 if they are homozygous for the major allele. The additive model counts the number of minor alleles for the gene (i.e., 0, 1, 2). Thus, those heterogeneous for the minor allele receive a 1 and those homogeneous for the allele received a 2. Finally, using the recessive model, individuals receive a score of 1 if they are homozygous for the minor allele and otherwise receive a score of 0. Among the 325 respondents, 2.50% were homozygous for the C allele (CC) at *rs2228145*, 20.00% were heterozygous (AC), and 77.50% were homozygous for the A allele (AA). Using the Hardy–Weinberg equilibrium test, the observed distribution of *rs2228145* did not differ significantly (chi-square = 2.255, *df* = 1, *p* = 0.133) from that predicted on the basis of simple Mendelian inheritance.

Studies have indicated that population genetic admixture may confound genetic findings. This study employed the Structure program version 2.3.4 [86] with a panel of 24 ancestry informative markers (AIMs) to infer the number of ancestral populations and to estimate an ancestry proportion of each participant. The average proportion of African ancestry in our sample is approximately 99%. There is no evidence for genetic admixture as a potential confounder in the present study.

#### 2.3.4. Control Variables

We control for a variety of neighborhood characteristics in order to reduce the probability that any effect of neighborhood disorder on inflammatory burden or physical health is due to its correlation with some other adverse dimension of community life. Further, some studies have found that the effects of neighborhood context on health outcomes are no longer significant after controlling for individual characteristics such as SES [47,87,88]. Therefore, our analyses include controls for several personal characteristics including SES, age, and exposure to childhood adversity.

*Concentrated disadvantage* was assessed with 2000 STF3A Census tract data. Following previous studies [89], the scale consisted of the following items: average per capita income, the percentage unemployed, percent of residents below the poverty threshold, the percentage of residents with less than a high school degree, the percentage of female-headed households, and the percentage of those receiving public assistance. To provide a weighted index for each item, per capita income was reverse-coded, and we used factor scores obtained through principal components analyses to form the scale. Coefficient alpha for the measure was 0.87.

*Residential stability* was measured using two items from the Census STF3A [89]: (1) the percentage of residents living in the same house over five years; and (2) the percentage of owner-occupied homes. The two items were standardized and summed. Coefficient alpha for the measure was 0.82.

*Neighborhood cohesion* was measured at Wave 5 using a revised version of the Social Cohesion and Trust Scale (PHDCN; [89]). The 15- item measure asks respondents to report the extent to which neighborhood residents trust and get along with each other, agree on values, are willing to help each other out, and care about what happens in their neighborhood. The scores were standardized and then summed to form a measure of neighborhood cohesion. Coefficient alpha for the scale was 0.90.

*Region* was coded 1 for respondents living in the south (67.4 percent) and 0 for those living in other areas of the country. Mean length of residence for respondents was nine years.

Family socioeconomic status (SES) was assessed using a composite measure based on the primary caregiver’s education and family income. These items were significantly correlated (*r* = 0.207, *p* < 0.001) and subsequently standardized and summed.

*Age*. At Wave 5, the respondents had a mean age of around 49 years, with a range of 35–65 years. Given this wide age range, age is included in all analyses.

*Childhood Adversity*. Past research has indicated that exposure to childhood adversity increases the chances of adult inflammation [39]. Therefore, our analyses control for childhood adversity using a four-item scale developed by Kessler [90]. Respondents reported whether or not they had experienced various traumas when they were growing up (e.g., Did you live with both your natural parents before your 16th birthday? While you were growing up, was anyone in your family violent toward another family member?). Scores were summed across items to form a measure of childhood adversity. The maximum possible score of four corresponded to subjects responding that they had experienced all of the different adversities. Approximately 73% of respondents reported they had experienced at least one childhood adversity when they were growing up.

### 2.4. Analytic Approach

Our data are hierarchically nested—that is, individual participants nested within neighborhoods. If samples were directly estimated by a general regression model, non-independent samples would overestimate the results [91]. To avoid this problem, we used a complex sampling design model available in the M*plus* 8.0 statistical software (TYPE = COMPLEX function, [92]). This model produced sandwich standard errors of the estimated coefficients that were adjusted for 88 Census tracts, thereby reducing Type 1 error inflation due to neighborhood clustering. This allowed the estimation of actual standard errors for clustered data in complex mediation models [93].

The measure of neighborhood disorder was standardized (mean of 0 and a standard deviation of 1) before interaction terms were calculated. Some advantages of using standardized scores in the interaction models include making coefficients easier to interpret and making the simple slope easier to test [94]. In addition, the variance inflation factors (VIF) and the tolerance statistics are used to detect whether multicollinearity exists among variables. Because inflammatory burden displayed a strong positive skew, it was transformed using log transformation to meet the assumption of linearity for ordinary least squares regression.

To test the hypotheses, ordered logistic regression with a complex sampling design was used for self-rated poor health status because this measure is an ordinal variable. Regarding self-reported health status, the first model was used to test for the main effect of neighborhood disorder. The second model included an inflammatory burden index to test the mediating model.

Turning to inflammatory burden, the studies included four regression models to test for the main effect of neighborhood disorder and the moderation effect of gene and neighborhood. Based upon the different coding schemes, the dominant genetic model was used in the first two models, and the additive model was used in the last two models. In these four models, Models 1 and 3 were used to test for main effects of neighborhood disorder, and Models 2 and 4 included the interaction terms necessary to test the moderating hypotheses. When the interaction effects were significant, post hoc analyses of interaction terms were conducted using the simple slope test and the proposed proportion of interaction (PoI) index [95]. Furthermore, the mediated moderation model [96] examines inflammatory burden as a possible mediator of the two-way interaction effect of neighborhood disorder and genotype on health status. The logic of the mediated moderation model is similar to traditional mediated models except that it focuses only on the relationships among an interaction term, mediator, and outcome rather than among other independent variables.

To test the stress-buffering hypothesis, the first model was used to test for the main effect of marital status. The second model included the interaction of neighborhood disorder with marital status. Finally, we added a three-way interaction to examine whether both marital status and genotype simultaneously moderate the effect of neighborhood disorder on inflammatory burden. To control for the inflated probability of Type I error in multiple tests, false discovery rates (FDR) *p* value was used. When interaction effects were present, post hoc analyses of significant interaction terms were conducted. Finally, we used the method available in M*plus* 8.0 to test the mediated moderation hypothesis [96] that the effect of *rs2228145* × neighborhood × marital status on self-rated health status is mediated by inflammatory burden.

## 3. Results

### 3.1. Descriptive and Association Analysis

Descriptive statistics for the study variables are presented in Table 1. A substantial proportion of participants reported that they were in poor/fair health status (Mean = 1.951, *SD* = 0.986). Four percent of respondents reported poor health and 28% indicated fair health status. Twenty-two percent of respondents scored greater than one standard deviation above the mean on the index of inflammatory burden. Approximately 15% of the respondents lived in neighborhoods with visible signs of disorder.

The correlation matrix is presented in Table 2. It shows a significant correlation between self-rated poor health and inflammatory burden, and both of these variables display significant associations with both neighborhood disorder and family SES. As expected, neighborhood disorder is significantly related to neighborhood disadvantage and cohesion, indicating that disordered neighborhoods are characterized by economic deprivation and a lack of social cohesion. Further, consistent with previous molecular genetic studies [67,68,69,70], Table 1 reveals that individuals carrying the C allele of *rs2228145* genotype demonstrate significantly higher levels of inflammatory burden (*r* = 0.217, *p* < 0.001). Importantly, studies have indicated that the presence of a gene–environment correlation (*r*GE) is likely to confound gene–environment interaction effects [56]. As shown in Table 2, there is no significant relationship between neighborhood disorder and the *rs2228145* genotype. This finding suggests the absence of an active *r*GE effect where individuals seek out neighborhoods that are compatible with their genetic predispositions. Furthermore, in analyses not shown, all associations of parent or child genotype with neighborhood disorder or health measures are nonsignificant, ruling out potential confounding effects of passive and evocative *r*GE attributable to *rs2228145* genotype selection into neighborhoods. Finally, consistent with previous studies [97,98], the table shows that being married is associated with higher socioeconomic status (SES). Thus, to account for variables that could provide plausible rival explanations, all analyses controlled for SES and demographic measures.

We did not use multilevel modeling in this study because it does not allow the testing of the mediated moderation effects, and approximately 40% of Census tracts have only one participant. Thus, to test the hypotheses and to correct for clustering bias, regression models with the COMPLEX option in M*plus* and robust maximum likelihood estimators are used in the following multivariate analyses.

### 3.2. The Effect of Neighborhood Disorder on Self-Rated Poor Health Status

Given that the measure of self-rated poor health is an ordinal variable, ordered logistic regression is used in Table 3. We first checked for potential multicollinearity among variables. VIF scores ranged between 1.053 for childhood adversity and 1.265 for length of residence, and all measures of tolerance were above 0.70, suggesting that multicollinearity is not identified (VIF < 10 and tolerance > 0.20). To understand the contributions of neighborhood disorder to self-rated poor health independent of individual and neighborhood socioeconomic status, we controlled for family socioeconomic status and various neighborhood characteristics in all models.

Model 1 presents the results of regressing self-rated poor health on observers’ ratings of neighborhood disorder and the control variables (See Table 3). As hypothesized, neighborhood disorder is related to self-rated poor health even after controlling for the demographic measures, childhood adversity, and other neighborhood characteristics. No variables other than family socioeconomic status and age show a significant effect, and childhood adversity presents a marginally significant effect. The results are consistent with numerous studies [12,13,63] reporting that residing in a disordered neighborhood has a deleterious effect on physical health even after controlling for socioeconomic status.

Model 2 adds the measure of inflammatory burden to the model. As predicted, inflammatory burden is positively and significantly related to self-rated poor health. Moreover, consistent with the mediation argument, the relationship between neighborhood disorder and self-reported health status is no longer significant when the measure of inflammatory burden is included in the model. Further, using the chi-square difference test, model fit is better when inflammatory burden is included (∆χ^2^ = 6.616, *df* = 1, *p* = 0.010).

To more stringently test the mediational model, we examined the relative strength of the direct and indirect pathways from neighborhood disorder to self-rated poor health. Using the Delta approach outlined by MacKinnon et al. [93], the indirect effect was significant (indirect effect = 0.018, *p* = 0.024) whereas the direct effect was not (direct effect = 0.106, *p* = 0.085). Approximately 15% of the variance in self-rated poor health explained by neighborhood disorder was accounted for by the measure of inflammatory burden.

### 3.3. The Effect of G × E Interaction on Inflammatory Burden

As an initial step in the gene–environment interaction analyses, a multicollinearity test was performed. The VIF ranged from 1.003 to 1.266, and all measures of tolerance were above 0.700, indicating that none of the models suffer from the problem of multicollinearity. As can be seen in Table 4, the dominant model for the genotype is used in Model 1 and Model 2. Therefore, *rs2228145* SNP is coded as a binary variable with the value 1 if individuals are either heterozygous or homozygous for the minor allele C and 0 for those homogeneous for the major A allele. Controlling for the demographic and control measures, Model 1 shows that neighborhood disorder and *rs2228145* are significantly associated with the inflammatory burden, suggesting that women living in disordered neighborhoods and carrying at least one minor allele have elevated levels of inflammation.

We then examined the moderating effect of variation at *rs2228145* by entering the interaction of neighborhood disorder and this genotype into the regression equation. As hypothesized, the results show that there is a significant interaction between neighborhood disorder and *rs2228145* in predicting inflammatory burden (b = 0.596, 95% CI (0.316, 0.876), *p* < 0.001, adjusted FDR *p* = 0.009). Furthermore, analysis using the simple slope test indicated that the slope for respondents with at least one copy of the minor C allele is significantly different from zero, whereas the slope is not significantly different from zero for those homozygous for the major allele.

**Figure 2:**
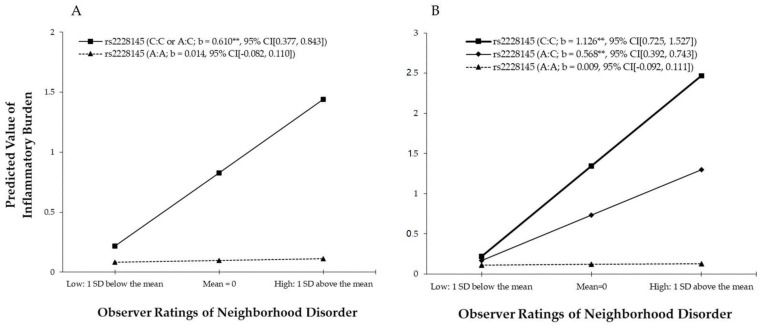
(**A**) The effect of observer ratings of neighborhood disorder on inflammatory burden by rs2228145. (**B**) The effect of observer ratings of neighborhood disorder on the inflammatory burden by rs2228145 (additive coding). Numbers in parentheses refer to simple slopes with 95% confidence intervals. ** *p* < 0.001.

To interpret the interaction of neighborhood disorder with *rs2228145* gene, we plotted estimated inflammatory burden at low (one standard deviation below the mean) and high (one standard deviation above the mean) levels of neighborhood disorder; the results are presented in Figure 2A. Consistent with our hypothesis, the fan-shaped pattern is shown. The figure demonstrates that the effect of neighborhood disorder on the inflammation responses is steeper for individuals who carry at least one copy of the minor C allele of *rs2228145* than for those who do not. Furthermore, based on the proposed proportion of interaction (PoI) index (Roisman et al. 2012 [95]; http://www.yourpersonality.net/interaction/ros.pl), the resulting value of 0.99 shows support for the diathesis–stress perspective in that individuals carrying the risk allele are at increased risk for inflammation in response to neighborhood disorder compared to those without the risk allele.

To explicate whether the effects of gene–environment interaction (G × E) are influenced by the method of coding SNPs, the additive model for the genotype is employed in Model 3 and Model 4. Unlike the dominant model, the additive model counts the number of minor alleles for the SNP (i.e., 0, 1, and 2; Lewis, 2002). As shown in Table 4, results from Model 3 and Model 4 are identical to results from Model 1 and Model 2. The findings suggest that the main effects of both neighborhood disorder and *rs2228145* SNP on the inflammatory responses are statistically significant. Model 4 then adds the multiplicative interaction term formed by multiplying neighborhood disorder by *rs22228145*. This interaction is significant (b = 0.558, 95% CI (0.325, 0.792), *p* < 0.001, adjusted FDR *p* = 0.005). Using the simple slope test, the results show that the slopes for respondents with either one copy or two copies of the minor C allele of *rs2228145* are significantly different from zero, whereas the slope is not significantly different from zero for those homozygous for the major allele.

As shown in Figure 2B, the graph of this interaction indicates a fan-shaped pattern identical to that depicted in Figure 2A. Further, similar to the findings for the dominant model, the PoI value for the additive model is 0.98, indicating that the model is also consistent with the diathesis–stress perspective. In other words, both the dominant and additive coding schemes tell a similar story. It should be noted that we also tested the recessive model for this SNP, whereby individuals receive a score of 1 if they are homozygous for the minor allele and otherwise receive a zero 0. Unfortunately, we only had eight (2.34%) participants which were homozygous for the minor C allele at this SNP. Thus, there was not enough statistical power to use the recessive model [99].

### 3.4. Mediated Moderation Effect of Inflammatory Burden

Next, the mediated moderation model [96] is tested to determine the extent to which the interaction of neighborhood disorder and genotype on self-rated poor health status is mediated by the inflammatory burden index. Using the Delta method, the results indicated that the indirect effect of G × E on self-rated poor health through inflammatory burden is significant for both the dominant model (indirect effect = 0.064, 95% CI (0.015, 0.113), *p* = 0.011) and the additive model (0.064, 95% CI (0.021, 0.107), *p* = 0.004) and accounts for approximately 28% of the total variance regardless of which approach is used to code for genetic risk. Consistent with our hypotheses, the combined effect of neighborhood disorder and *rs2228145* minor C allele has a significant indirect effect on self-rated poor health through its effect on inflammatory burden.

### 3.5. Test of the Stress-Buffering Hypothesis

The next set of models examines the hypotheses that both marital status and *rs2228145* gene moderate the effect of neighborhood disorder on inflammatory burden; the results are presented in Table 5. Model 1 shows that neighborhood disorder is positively and significantly associated with inflammatory burden, whereas the direct effect of marital status on the inflammatory burden is insignificant, indicating that marriage as a source of support does not directly reduce inflammatory distress. Model 2 adds the interaction of marital status with neighborhood disorder. The interaction is also insignificant.

The last model in Table 5 incorporates a three-way interaction of neighborhood disorder × *rs2228145* × marital status to test the hypothesis that marital status buffers the impact of neighborhood disorder and genotype on inflammatory burden. As expected, the findings reveal that there is a significant three-way interaction effect (b = −0.529, 95% CI (−0.967, −0.091), *p* = 0.018, adjusted FDR *p* = 0.010). It should also be noted that family structure might be confounded with family SES [100]. Instead of marriage status, we also examined a three-way interaction model using family SES. The findings showed that the moderating effect of family SES was insignificant, suggesting that marriage as a source of social and emotional support is not equal to family SES.

**Figure 3:**
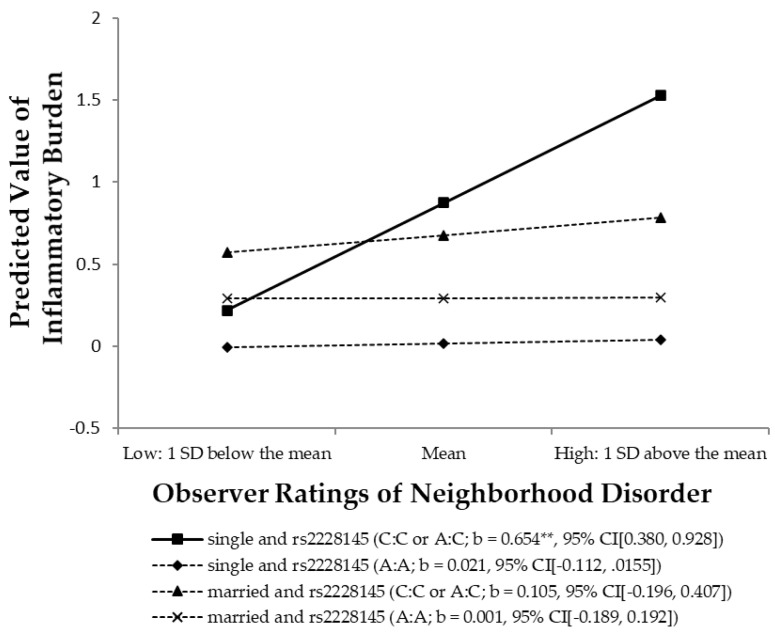
The Effect of Observer Ratings of Neighborhood Disorder on the Inflammatory Burden by *rs2228145* and Marital Status. Numbers in parentheses refer to simple slopes with 95% confidence intervals. ** *p* < 0.001.

To further examine these relationships, we graphed the effect in Figure 3 for levels of neighborhood disorder that range from 1 standard deviation (SD) below to 1 SD above the mean. As can be seen in the figure, when individuals live in disordered neighborhoods, those with at least one copy of the minor C alleles on *rs2228145* show the highest level of inflammation, but such an effect is only evident for those in the sample who are not married. Based on a simple slope test, the results suggest that the regression line depicting the relationship between neighborhood disorder and the inflammatory burden is significantly steeper for individuals with at least one copy of the minor alleles on *rs2228145* and unmarried than for married women or for women without this minor allele on *rs2228145*. Among the 325 respondents used in our analysis, approximately 15% have at least one copy of the minor allele on *rs2228145* and are unmarried.

Finally, a simple slope difference test, presented in Figure 3, is used to test which of the slopes statistically differ from each other [94]. As expected, the results show that unmarried women with the risk allele of *rs2228145* gene are more vulnerable to neighborhood contexts than others, whereas none of the simple slopes are significantly different among the three groups: married women without the risk allele, married women with the risk allele, and unmarried women without the risk allele.

Although not presented for the purpose of brevity, the results using the additive coding of *rs2228145* (2 = C/C; 1 = A/C; 0 = A/A) are almost identical to the results using the dominant coding schemas. Therefore, findings from the current study suggest that marriage is particularly helpful for individuals carrying genetic risk and living in disordered neighborhoods. Finally, using the mediated moderation model with the Delta method, the indirect effect of G × neighborhood × marital status on self-reported health status through the inflammatory burden is significant (indirect effect = −0.067, 95% CI (−0.130, −0.004), *p* = 0.038) and accounts for 25.77% of the total variance. These findings support the hypothesis that the interaction effect of neighborhood disorder, the *rs2228145* gene, and marital status has an indirect effect on self-rated poor health through its effect on inflammatory burden.

## 4. Discussion

Myriad studies have reported that social stress is related to illness and health [20]. Much of this research has been guided by the stress process model [18,19,34], which posits that prolonged exposure to stressful conditions activates a physiological stress response that increases the chances of tissue damage, impaired immune functioning, and illness. In large measure, studies regarding the link between stress and illness have focused upon personal stressors. Recently, however, researchers have begun to investigate the extent to which adverse neighborhood conditions might also increase the risk for disease. A popular hypothesis is that it is the ambient threat posed by social and physical disorder (e.g., graffiti, scattered trash, vandalism, abandoned buildings, public drinking, petty crime), and not simply neighborhood disadvantage, that is highly stressful and leads to illness [12,13,14,15,16]. Consonant with this idea, several studies have reported an association between neighborhood disorder and various health outcomes [12,14,16,28]. Although there is evidence that other dimensions of neighborhood adversity, such as poverty or neighborhood SES [101,102], are also related to health, the social disorder model suggests that these relationships would not have been significant had these studies included a measure of neighborhood physical disorder.

The present study was an attempt to overcome some of the methodological and theoretical limitations that have plagued past studies of the link between neighborhood physical disorder and health. Our goal was to utilize more objective measures of both neighborhood characteristics and illness, and to expand the basic social disorder model in an effort to make it more biologically integrated. Specifically, we extended prior research in the following ways:

First, most studies employ self-ratings of social disorder as well as self-ratings of health. As a consequence, associations reported between neighborhood disorder and illness are undoubtedly inflated due to shared methods variance [17]. We avoided this problem in the present study by using ratings by trained observers to assess neighborhood disorder [36]. Our results indicated that observer ratings of disorder were significantly associated with respondents’ global ratings of their health, and this association held after controlling for various individual-level factors as well as other dimensions of neighborhood disadvantage and social isolation.

Second, while the stress process model posits that heightened physiological stress serves as the mechanism that links adverse conditions to illness, potential mediating physiological factors are rarely assessed. This is particularly true of neighborhood studies. The present study tested the hypothesis that chronic systemic inflammation is the physiological mechanism that mediates much of the impact of neighborhood disorder on health. This expectation was based upon the fact that biomarkers of systemic inflammation are an important signal of immune dysfunction that leads to changes in major physiological systems that portend the development of the age-related diseases that kill most individuals in Western societies [40]. Further, there is strong evidence that exposure to stress is associated with elevated inflammation. As predicted, we found that biomarkers of inflammatory burden mediated much of the association between neighborhood disorder and global ratings of health. Indeed, the association between neighborhood disorder and self-rated health was no longer significant once inflammatory burden was entered into the model.

These analyses enhance our understanding of the nature or meaning of global ratings of health. Researchers have not known what to make of such measures. On the one hand, there is the danger that they may be contaminated by psychological factors such as personality and mood. On the other hand, self-ratings of health have been shown to be stronger predictors of mortality than diagnosed illness [51,52]. This robust finding between perceived health and mortality has been perplexing. Recently, however, Christian et al. [25] argued that elevated levels of inflammation likely give rise to decreased appetite, fatigue, and feelings of not being well. Consonant with this argument, they found that biomarkers of inflammation (IL-6 and CRP) were significantly related to global ratings of health. Similar to Christian et al. [25], we found a significant association between inflammatory burden and self-rated health. Our results extend their finding by showing that this relationship explains much of the association between stressful conditions such as neighborhood disorder and global ratings of health.

Although we found significant relationships between neighborhood disorder and both inflammatory burden and self-rated health, the magnitude of these associations, as expected, was modest. Thus, a major concern of the present paper was to test hypotheses regarding the extent to which genetic variability and access to social support might moderate the link between neighborhood disorder and our measures of health. As predicted, allelic variation at the IL-6r gene (A > C, *rs2228145*), a gene that has been shown to influence inflammation, amplified the relation between exposure to neighborhood disorder and inflammatory burden. Moreover, findings from the mediated moderation model indicated that neighborhood disorder and genotype interact to influence self-rated health through their interaction effect on inflammatory burden. These results provide important evidence that the health consequences of neighborhood conditions may not be the same for all people. Our finding suggests that carriers of the minor C allele of the IL-6r gene are significantly more likely to respond to neighborhood disorder with increased inflammation and self-perceptions of poor health.

We also examined whether marital status might buffer the negative health effects of neighborhood disorder. A wealth of studies have shown that married individuals tend to show better health than single persons, and that marriage often operates as a buffer against the probability that stress will lead to illness [20]. Our results indicate that marital status cannot directly buffer this effect, but it can moderate the untoward health consequences of genetic risk. Only carriers of the minor C allele and who were also single showed elevated inflammation and poorer perceived health when residing in a disordered neighborhood. The results suggest that although individuals with the minor allele of the IL-6r gene were at increased risk for illness in response to neighborhood disorder, this effect was eliminated for married respondents.

While our study was able to extend past research in several respects, it also suffered from various limitations. Chief among them is the fact that our sample was limited to African American women. Thus, it did not allow us to test for gender differences. In some respects, however, this shortcoming might be seen as a strength. Myriad studies have established that African Americans are at higher risk than other ethnic groups for chronic illness [5,33], for inflammatory burden [29,30], and for exposure to neighborhood disorder [32]. Further, there is evidence indicating that the impact of neighborhood characteristics on health is more pronounced for women than men [12,28,31]. All of this argues for research investigating the effect of neighborhood conditions on the health of African American women. Nevertheless, clearly there is a need to replicate our findings with samples that are more ethnically diverse and that include males.

A second limitation that deserves to be mentioned is our use of marital status as an indicator of social support. Past research suggests that, in most cases, quality of romantic relationship is a better measure of support than simply marital status. Thus, for example, married persons in unhappy relationships tend to show more health problems than unmarried persons [20], and warm and supportive cohabiting relationships often provide health benefits similar to those obtained from a happy marriage [103]. All of this suggests that the buffering effect of a romantic partner found in the present study would have been even more powerful if we had been able to assess levels of support from a romantic partner rather than simply marital status. However, it may be that when women are feeling vulnerable given dangerous environments, simply sharing residence with a male enhances their sense of safety and security [104,105]. The crime and incivilities that take place in an area tend to be committed by males and women may feel less threatened by these individuals when they live with a man. To the extent that this is true, both marriage and cohabitation should reduce the probability that neighborhood disorder will lead to physiological stress and poor health, and quality of relationship should have little impact beyond co-residential status. Further, if this is the mechanism that accounts for our findings, both the health consequences of neighborhood disorder and the buffering effect of a romantic partner are likely less for males than females. Men would be less likely to feel threatened by neighborhood disorder and hence would be less likely to respond to such conditions with inflammation and poor health. Moreover, to the extent that an association between ambient threat and poor health also exists for males, having a female romantic partner may do little to enhance feelings of safety. Unfortunately, it was not possible to test these various ideas in the present study. Hopefully, subsequent studies can tease out whether it is relationship status or quality that furthers health in a dangerous neighborhood and whether this buffering effect differs by gender.

In conclusion, while our study suffered from certain constraints, it also extended prior research in a number of respects. In addition to proffering more convincing evidence of an association between social disorder and illness, we identified inflammatory burden as the mediating biophysiological mechanism that links stressful neighborhood conditions to perceived health. Indeed, our findings provided added support regarding the reason that global self-ratings of health are such good predictors of mortality: they are indicators of chronic, systemic inflammation. Finally, our analyses indicated that both genetic variation and marital status serve to moderate the association between neighborhood physical disorder and health. Overall, our findings suggest the importance of constructing integrated models that bring together social and biological variables. By specifying biophysiological mechanisms (inflammation, genetic variability) that link social conditions (neighborhood disorder, social support) to illness, our sociological models become more comprehensive and precise. They also become more useful and compelling for medical and public health professionals concerned with designing social policies and preventive interventions to enhance health.

## Figures and Tables

**Table 1:** Descriptive statistics for study variables (*N* = 325).

Variables	Mean (*SD*)	%
Self-rated poor health	1.95 (0.99)	
Inflammatory burden	−0.05 (1.45)	
Neighborhood disorder	0.00 (1.00)	
*rs2228145* (1 = C:C or A:C), %		22.50%
Married, %		30.50%
Childhood adversity	1.46 (1.24)	
Family socioeconomic status	0.00 (0.78)	
Age	46.17 (6.10)	
South, %		67.40%
Length of residence	8.52 (8.59)	
Neighborhood cohesion	−0.38 (9.61)	
Neighborhood disadvantage	0.08 (1.04)	
Residential stability	0.04 (1.00)	

**Table 2:** Correlation matrix for the study variables.

	1	2	3	4	5	6	7	8	9	10	11	12	13
1. Self-rated poor health	⸻												
2. Inflammatory burden	0.11 *	⸻											
3. Neighborhood disorder	0.11 *	0.11 *	⸻										
4. *rs2228145* (1 = C:C or A:C)	−0.07	0.22 **	−0.01	⸻									
5. Married	−0.12 *	0.11 ^†^	−0.04	0.06	⸻								
6. Childhood adversity	0.08	−0.01	−0.06	0.03	−0.03	⸻							
7. FSES	−0.17 **	0.11 *	−0.03	0.03	0.31 **	0.03	⸻						
8. Age	0.10 ^†^	0.14 *	−0.03	0.01	0.01	0.03	0.04	⸻					
9. South	−0.02	−0.13 *	−0.16 **	−0.02	−0.01	−0.05	−0.07	−0.10 ^†^	⸻				
10. Length of residence	0.01	0.07	0.05	−0.02	0.09 ^†^	−0.11 ^†^	0.10 ^†^	0.32 **	0.14 *	⸻			
11. Neighborhood cohesion	−0.04	0.06	−0.10 *	0.02	0.05	−0.15 **	0.10 ^†^	0.11 *	0.16 **	0.22 **	⸻		
12. ND ^a^	0.10 ^†^	−0.10	0.15 **	−0.03	−0.14 *	0.03	−0.24 **	−0.14 *	−0.19	−0.07	−0.20 **	⸻	
13. Residential stability ^a^	−0.07	0.13 ^†^	−0.01	0.01	0.14 **	−0.13 *	0.10	0.09	0.17	0.25 **	0.14 **	−0.25	⸻

^†^*p* ≤ 0.10; * *p* ≤ 0.05; ** *p* ≤ 0.01 (two-tailed tests); *N* = 325; *Note*: ND = Neighborhood disadvantage; FSES = Family socioeconomic status; ^a^ Robust standard error was used.

**Table 3:** Ordered logistic regression of self-rated poor health status on observer ratings of neighborhood disorder and inflammatory burden.

*Dependent variable:*	Model 1 (ND)		Model 2 (ND, IB)	
Self-rated poor health status (0–4)	*Coeff.* (SE)	Odds Ratio	*Coeff.* (SE)	Odds Ratio
*Independent variable*				
Observer ratings of neighborhood disorder (ND)	0.123 *	1.131	0.109 †	1.115
	(0.058)		(0.058)	
*Mediator*				
Inflammatory burden (IB)			0.094 **	1.099
			(0.037)	
*Control variables*				
Childhood adversity	0.074 †	1.077	0.073 †	1.076
	(0.041)		(0.041)	
Family SES	−0.215 **	0.807	−0.228 **	0.796
	(0.072)		(0.076)	
Age	0.023 *	1.023	0.021 †	1.021
	(0.011)		(0.011)	
South	0.045	1.046	0.086	1.090
	(0.173)		(0.177)	
Length of residence	−0.001	0.999	−0.001	0.999
	(0.009)		(0.009)	
Neighborhood cohesion	0.000	1.000	−0.001	0.999
	(0.006)		(0.007)	
Concentrated disadvantage	0.050	1.051	0.060	1.062
	(0.054)		(0.055)	
Residential stability	−0.047	0.954	−0.063	0.939
	(0.058)		(0.063)	
Thresholds				
Threshold parameter 0–1	−1.434 **	0.238	−1.424 **	0.241
	(0.178)		(0.180)	
Threshold parameter 1–2	−0.325 *	0.723	−0.306 †	0.736
	(0.158)		(0.158)	
Threshold parameter 2–3	0.590 **	1.804	0.619 **	1.857
	(0.155)		(0.157)	
Threshold parameter 3–4	1.976 **	7.214	2.015 **	7.501
	(0.178)		(0.177)	
*R*-Square	0.072		0.088 *	
∆*R*-Square			0.016 **	

Notes: Unstandardized coefficients are shown with robust standard errors in parentheses; SES, age, neighborhood cohesion, concentrated disadvantage, and residential stability are centered by mean subtraction; observer ratings of neighborhood disorder are standardized by z-transformation (mean = 0 and *SD* =1); *N* = 325. † *p* ≤ 0.100; * *p* ≤ 0.050; ** *p* ≤ 0.010 (two-tailed tests).

**Table 4:** Regression of inflammatory burden on observer ratings of neighborhood disorder and *rs2228145.*

*Dependent variable:*	Model 1 (ND, Gene)		Model 2 (ND × Gene)		Model 3 (ND, Gene)		Model 4 (ND × Gene)	
Log-transformed Inflammatory burden	b	*β*	b	*β*	b	*β*	b	*β*
*Environment and Genetic Variables*								
Observer ratings of neighborhood disorder (ND)	0.164 *	0.114	0.014	0.010	0.163 *	0.112	0.009	0.006
	(0.074)		(0.049)		(0.075)		(0.052)	
*rs2228145* (1 = C:C or A:C) (Gene)	0.727 **	0.210	0.731 **	0.211				
	(0.197)		(0.182)					
*rs2228145* (2 = C:C; 1 = A:C; 0 = A:A) (Gene)					0.633 **	0.213	0.612 **	0.206
					(0.225)		(0.217)	
*Gene-Environment Interaction*								
Neighborhood disorder			0.596 **	0.212				
× *rs2228145* (1 = C:C or A:C)			(0.143)					
Neighborhood disorder							0.558 **	0.223
× *rs2228145* (2 = C:C; 1 = A:C; 0 = A:A)							(0.119)	
*Control Variables*								
Childhood adversity	0.012	0.011	−0.003	−0.002	0.008	0.007	−0.003	−0.003
	(0.063)		(0.059)		(0.064)		(0.060)	
Family SES	0.115	0.061	0.140 †	0.075	0.112	0.060	0.139 †	0.075
	(0.081)		(0.083)		(0.082)		(0.084)	
Age	0.023 †	0.097	0.024 †	0.102	0.022 †	0.092	0.024 †	0.102
	(0.013)		(0.013)		(0.013)		(0.013)	
South	−0.420 *	−0.136	−0.404 *	−0.131	−0.402 *	−0.130	−0.394 *	−0.128
	(0.174)		(.0180)		(0.172)		(0.178)	
Length of residence	0.001	0.006	−0.001	−0.004	−0.001	−0.001	−0.003	−0.018
	(0.008)		(0.007)		(0.008)		(0.007)	
Neighborhood cohesion	0.006	0.043	0.011	0.076	0.006	0.043	0.012	0.078
	(0.009)		(0.009)		(0.009)		(0.009)	
Concentrated disadvantage	−0.104	−0.075	−0.107	−0.077	−0.104	−0.074	−0.103	−0.074
	(0.100)		(0.103)		(0.099)		(0.101)	
Residential stability	0.169 †	0.117	0.172 †	0.119	0.174 †	0.120	0.179 †	0.123
	(0.094)		(0.094)		(0.094)		(0.094)	
*Constant*	0.065	0.045	0.098	0.068	0.074	0.051	0.122	0.084
	(0.160)		(0.161)		(0.164)		(0.168)	
*R*-Square	0.122		0.153		0.123		0.159	
∆*R*-Square			0.031 **				0.036 **	

Notes: Unstandardized and standardized coefficients are shown with robust standard errors in parentheses; SES, age, neighborhood cohesion, concentrated disadvantage, and residential stability are centered by mean subtraction; observer ratings of neighborhood disorder (ND) are standardized by z-transformation (mean = 0 and *SD* =1); *N* = 325. † *p* ≤ 0.100; * *p* ≤ 0.050; ** *p* ≤ 0.010 (two-tailed tests).

**Table 5:** Regression analysis examining the relationships among neighborhood disorder, *rs2228145*, marital status, and inflammatory burden.

*Dependent variable:*	Model 1 (ND, married)		Model 2 (ND × married)		Model 3 (ND × married × Gene)	
Log-transformed Inflammatory burden	b	*β*	b	*β*	b	*β*
*Environment and Genetic Variables*						
Observer ratings of neighborhood disorder (ND)	0.167 *	0.115	0.215 *	0.148	0.021	0.015
	(0.085)		(0.089)		(0.068)	
People married or single (1 = married)	0.216	0.069	0.212	0.067	0.277	0.088
	(0.169)		(0.177)		(0.207)	
*rs2228145* (1 = C:C or A:C)					0.856 **	0.247
					(0.292)	
*Gene-Environment Interaction*						
Neighborhood disorder × Married			−0.220	−0.072	−0.020	−0.006
			(0.186)		(0.135)	
Neighborhood disorder × *rs2228145*					0.633 **	0.225
					(0.167)	
People married or single × *rs2228145*					−0.472	−0.089
					(0.385)	
Neighborhood disorder × Married × *rs2228145*					−0.529 *	−0.066
					(0.224)	
*Control Variables*						
Childhood adversity	0.020	0.017	0.016	0.014	0.001	0.001
	(0.067)		(0.064)		(0.058)	
Family SES	0.085	0.046	0.079	0.043	0.097	0.052
	(0.079)		(0.108)		(0.090)	
Age	0.023 †	0.099	0.024 †	0.101	0.024 †	0.100
	(0.013)		(0.014)		(0.013)	
South	−0.428 *	−0.139	−0.437 *	−0.142	−0.428 *	−0.139
	(0.184)		(0.176)		(0.186)	
Length of residence	−0.001	−0.002	−0.001	−0.004	0.001	0.004
	(0.008)		(0.010)		(0.008)	
Neighborhood cohesion	0.007	0.047	0.007	0.043	0.010	0.067
	(0.009)		(0.009)		(0.009)	
Concentrated disadvantage	−0.106	−0.076	−0.108	−0.077	−0.104	−0.075
	(0.104)		(0.082)		(0.103)	
Residential stability	0.163 †	0.113	0.157	0.109	0.165 †	0.114
	(0.096)		(0.083)		(0.093)	
*Constant*	0.169	0.117	0.182	0.126	0.017	0.012
	(0.179)		(0.194)		(0.188)	
*R*-Square	0.082		0.086		0.163	
∆*R*-Square			0.004		0.077 **	

Notes: Unstandardized and standardized coefficients are shown with robust standard errors in parentheses; SES, age, neighborhood cohesion, concentrated disadvantage, and residential stability are centered by mean subtraction; observer ratings of neighborhood disorder are standardized by z-transformation (mean = 0 and *SD* =1); *N* = 325. † *p* ≤ 0.100; * *p* ≤ 0.050; ** *p* ≤ 0.010 (two-tailed tests).

## Data Availability

The data presented in this study are available on request from the corresponding author. The data are not publicly available.
